# Editorial: Impacts of metal and xenobiotic-induced stress on antibiotic resistance in microbial communities

**DOI:** 10.3389/fmicb.2025.1745065

**Published:** 2025-12-05

**Authors:** Rojana Sukchawalit, Agata Goryluk-Salmonowicz, Jon L. Hobman, Magdalena Popowska

**Affiliations:** 1 Laboratory of Biotechnology, Chulabhorn Research Institute, Bangkok, Thailand; 2 Faculty of Biology and Biotechnology, Department of Biochemistry and Microbiology, Institute of Biology, Warsaw University of Life Sciences, Warsaw, Poland; 3 School of Biosciences, The University of Nottingham, Sutton Bonington Campus, Sutton Bonington, United Kingdom; 4 Faculty of Biology, Department of Bacterial Physiology, Institute of Microbiology, University of Warsaw, Warsaw, Poland

**Keywords:** antimicrobial resistance (AMR), anthropogenic stressors, microbiome, metals, microplastic, co-selection, horizontal gene transfer (HGT), AMR mitigation strategies

Antimicrobial resistance (AMR) is recognized by the World Health Organization (WHO) as one of the most serious global threats to public health and sustainable development. Although antibiotic misuse and overuse remain the primary drivers, awareness is growing that other anthropogenic stressors—such as toxic and essential metals, biocides, disinfectants, and xenobiotics—also play a crucial role in promoting resistance. These substances are widely used in medicine, agriculture, and industry, and often enter the environment through wastewater or surface runoff, where they can co-select for resistant bacteria and resistant genes even in the absence of antibiotics.

The environmental dimension of AMR has long been overshadowed by clinical research but is now recognized as integral to the One Health concept, which links human, animal, and ecosystem health. Metals and non-antibiotic antimicrobials can act as selective agents that maintain or amplify resistance determinants, which are frequently located on mobile genetic elements (MGEs) that facilitate horizontal gene transfer. Similarly, pollutants such as microplastics can adsorb antibiotics and metals, creating microhabitats that promote biofilm formation and the persistence of resistant taxa. Understanding the mechanisms by which these pollutants influence microbial communities and resistomes is essential for predicting the emergence of AMR across natural and engineered ecosystems.

## Analysis of the Research Topic contributions

This Research Topic brings together six studies that explore how metals, microplastics, and other xenobiotics shape microbial evolution and resistance in various One Health environments, from alpine waters and Arctic human microbiomes to agricultural soils and gut microbiomes.

One of the emerging contaminants that interacts synergistically with antibiotics to promote AMR is microplastics. Mosca Angelucci et al. examined how ciprofloxacin and polyethylene terephthalate (PET) microplastics jointly alter microbial communities in glacier-fed alpine spring water. Their microcosm experiments demonstrated that microplastics adsorb ciprofloxacin and enhance biofilm formation, creating favorable niches for resistant bacteria. Combined exposure (CIP + PET) increased the copy numbers of plasmid-mediated quinolone resistant genes *qnrA* and *qnrB*, and selected for taxa such as *Achromobacter* and *Luteolibacter*. Although ciprofloxacin alone was the dominant selective force, microplastics amplified its effects by stabilizing biofilms and facilitating horizontal gene transfer.

The combined exposure of two factors promoting AMR was also examined by Suprenant et al.. The researchers explored how zinc exposure alters the trajectory of ciprofloxacin resistance in *E. coli*. Pre-exposure to zinc sulfate for 5 days accelerated subsequent resistance development, yielding MICs up to 4 × higher after short-term antibiotic exposure. Interestingly, when zinc and ciprofloxacin were present simultaneously, resistance emergence was delayed. Whole-genome and RNA sequencing revealed no stable mutations, indicating transcriptional or metabolic regulation rather than genetic fixation. This work demonstrates that essential metals, such as zinc, which are used in supplements (chemical fertilization) or are prevalent in contaminated environments, can transiently modulate bacterial physiology, altering how resistance evolves under antibiotic pressure—a crucial insight for both public health and environmental management.

More extensive research regarding this Research Topic was conducted by Szadziul et al.. The authors analyzed how the physico-chemical properties of Polish agricultural and forest soils shape ARG and MGE abundance. Using high-throughput qPCR, they quantified 27 clinically relevant ARGs and five MGEs, finding significantly higher levels in cultivated soils, especially those that were manured, compared with forests. Concentrations of heavy metals, notably mercury, along with arsenic, chromium, and zinc, correlated positively with ARG abundance, while sandy texture and high C:N ratios correlated negatively. These results highlight that soil chemistry and contamination profiles strongly influence resistome structure and that heavy metal co-selection operates as an important environmental mechanism that sustains AMR outside of clinical contexts.

The co-selection mechanism as an AMR-promoting factor was also examined by Hauptmann et al.. The researchers investigated the gut microbiomes of Indigenous Greenlanders, whose traditional marine-based diets contain elevated levels of mercury and cadmium. Multi-omics analyses identified near-complete genomes of mercury-resistant bacteria that express the *merA* and *merB* genes responsible for detoxifying Hg. These genes co-occurred with antibiotic resistance determinants on shared MGEs, indicating genomic linkage between heavy metal and antibiotic resistance. While overall diversity metrics were stable, the persistence of co-resistant strains in a population with low antibiotic use illustrates that chronic environmental exposure to metals alone can sustain AMR reservoirs. This study provides direct human evidence of environmental–microbial co-selection and exemplifies the One Health interplay between diet, pollution, and resistance. Similarly, Pagano et al. focused on the influence of metals on gut microbial consortia. They examined the influence of different iron forms (hemin vs. free Fe^2+^/Fe^3+^) on amoxicillin susceptibility in *ex vivo* gut microbial consortia. Using long-read 16S sequencing, the authors achieved ~77% species retention over 24 h and found that hemin, but not free iron, protected *Bacteroides, Escherichia–Shigella*, and *Parabacteroides* from amoxicillin. These findings suggest that iron speciation modulates antibiotic activity through metabolic interactions and redox balance in complex communities. The work underscores how host- or diet-derived metals may influence treatment efficacy and resistance selection within the intestinal microbiome.

Integrating environmental monitoring of metal content into AMR policies is essential for land-based climate change mitigation strategies. A promising strategy was presented by Demissie et al. investigated how alkalization and UV/H_2_O_2_ oxidation inactivate antibiotic-resistant *Escherichia coli* and *Enterococcus faecium*, and degrade their respective resistant genes (*blaCTX–M* and *vanA*) in source-separated urine. Treatments at pH 10.8–12.5 achieved more than 7 log_10_ reductions in bacterial counts and up to 3 log_10_ reductions in gene copies. Importantly, simple alkaline storage for 3 h at pH 12.5 achieved similar effects to energy-intensive UV/H_2_O_2_ exposure, offering a practical, low-cost solution for safe nutrient recycling. By coupling hygienization with resistant gene removal, the study demonstrates that engineering design can directly contribute to AMR mitigation and circular resource use—a key operationalization of One Health principles in sanitation and waste valorization.

## Synthesis and outlook

**Figure 1:**
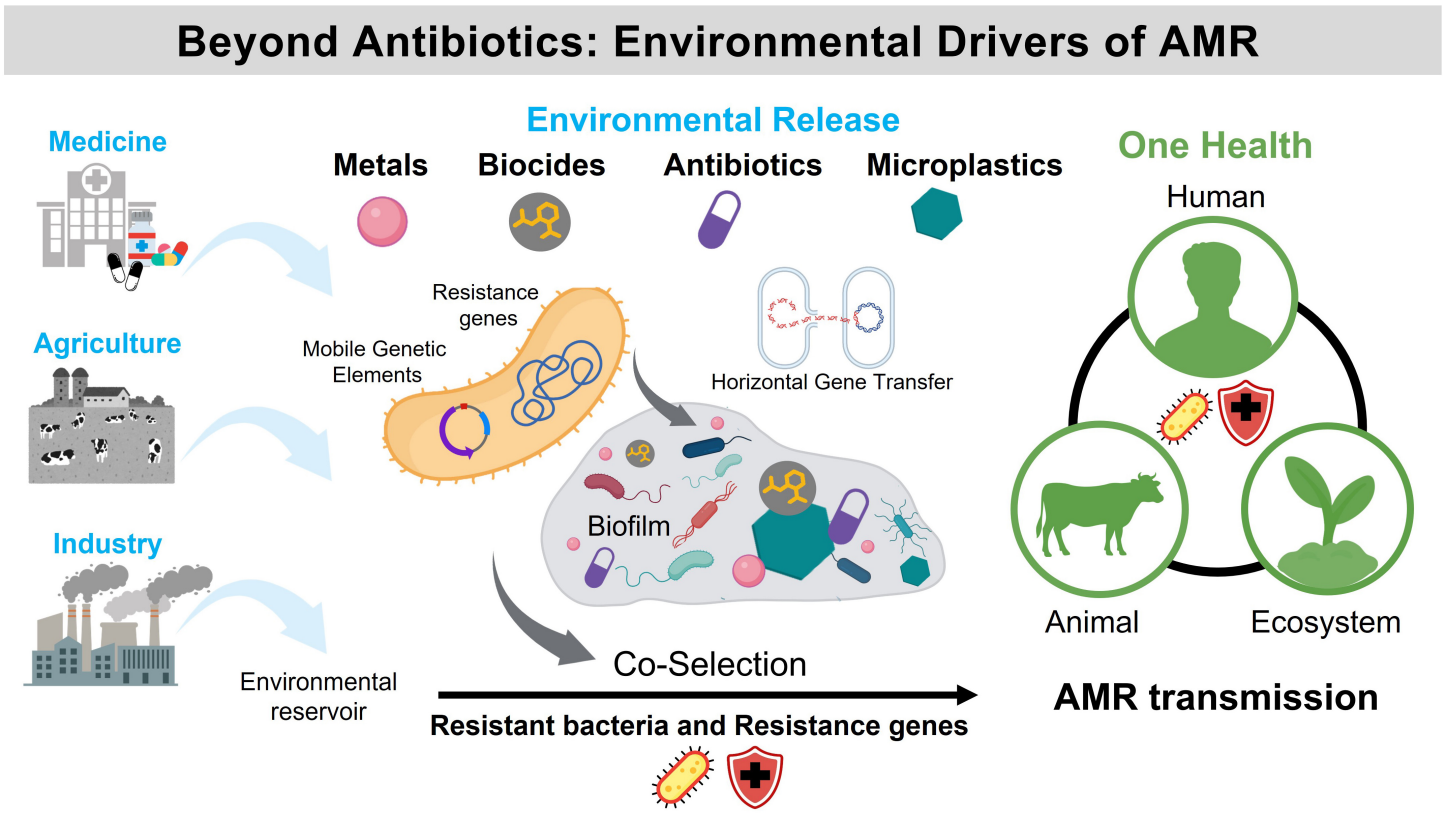
Environmental drivers of antimicrobial resistance (AMR).

Together, these studies reveal a continuum of processes by which metals and xenobiotics shape microbial resistance from biochemical modulation to ecological dissemination (Figure 1). They collectively demonstrate that:

- Metals and synthetic pollutants act as selective agents that maintain and amplify ARGs even in the absence of antibiotic exposure (Szadziul et al.; Hauptmann et al.).
- Synergistic or sequential interactions between contaminants such as antibiotics with microplastics or pre-exposure to zinc—can accelerate the evolution of resistance (Mosca Angelucci et al.; Suprenant et al.).
- Environmental matrices, including soil, freshwater, and waste streams, are active sites of co-selection and horizontal gene transfer (Mosca Angelucci et al.; Demissie et al.).
- Essential metals, such as iron and zinc, can either enhance or mitigate the effects of antibiotics, depending on their chemical speciation and exposure sequence (Pagano et al.; Suprenant et al.).
- Human populations distant from intensive antibiotic use can harbor co-resistant microbiota due to dietary or environmental exposure to metals (Hauptmann et al.).

By spanning molecular, environmental, and human dimensions, this Research Topic emphasizes that AMR is not solely a product of antibiotic misuse but an ecological consequence of multiple, interacting chemical pressures. Effective mitigation, therefore, requires regulatory attention to non-antibiotic antimicrobials and pollutants, alongside antibiotic stewardship. Integrating environmental monitoring, pollution control, and microbial ecology into AMR policy frameworks will be vital to operationalizing the One Health approach and preventing resistance propagation across interconnected ecosystems.

Antibiotic misuse and overuse are the main causes of AMR. However, metals and xenobiotic-induced stressors—such as biocides and microplastics—also play a critical role in its emergence and persistence. These agents, extensively applied in medicine, agriculture, and industry, are released into the environment, where they contribute to selective pressure. Resistant genes, which are frequently located on MGEs that facilitate horizontal gene transfer, can be maintained or promoted by the presence of metals and non-antibiotic antimicrobials that act as co-selective agents. Microplastics further exacerbate the problem by adsorbing antibiotics, metals, and other xenobiotics, creating microhabitats that promote biofilm formation and the persistence of resistant taxa. Environmental–microbe interactions thus support the co-selection of resistant bacteria and resistant genes, even in the absence of antibiotics. The One Health framework emphasizes the interconnectedness of human, animal, and environmental health in shaping the emergence and dissemination of AMR.

